# Individualized perioperative decision-making for intraoperative cell salvage in an Rh(D)-negative patient with placenta percreta: a case report

**DOI:** 10.3389/fmed.2026.1874079

**Published:** 2026-09-30

**Authors:** Yanping Lu, Xuewei Wang, Cuiyuan Huang, Henjing Zou, Changhui Shao, Shan Ou

**Affiliations:** 1 Department of Anesthesiology, Chengdu Integrated TCM& Western Medicine Hospital, Chengdu, China; 2 Department of Emergency, Chengdu Second People’s Hospital, Chengdu, China

**Keywords:** case report, intermittent abdominal aortic balloon occlusion, intraoperative cell salvage, obstetric anesthesia, patient blood management, placenta accreta spectrum, placenta percreta, Rh(D)-negative

## Abstract

Placenta accreta spectrum (PAS), and particularly placenta percreta, is one of the most hemorrhage-prone conditions in obstetric surgery. In Rh(D)-negative women, the conventional toolkit of patient blood management is constrained: compatible allogeneic supply is scarce, and intraoperative cell salvage (ICS) raises a specific concern about residual fetal red cell exposure and Rh alloimmunization. We report the perioperative course of a 29-year-old gravida 3, para 2 woman with O Rh(D)-negative blood, preoperative anemia (hemoglobin 86 g/L), placenta percreta with suspected bladder invasion, and intrahepatic cholestasis of pregnancy, who underwent cesarean subtotal hysterectomy at 32 weeks of gestation after multidisciplinary planning. Hemorrhage control combined intermittent abdominal aortic balloon occlusion (IABO), tranexamic acid, definitive surgical hemostasis, and timely transfusion of 7 units of cross-matched O Rh(D)-negative packed red blood cells and 600 mL of fresh frozen plasma. ICS was activated after fetal delivery and 900 mL of processed autologous blood was held in reserve; reinfusion was not undertaken after hemorrhage had been controlled through IABO, definitive surgical hemostasis, and same-type allogeneic transfusion, while hemoglobin remained ≥ 80 g/L and no additional red-cell support was considered clinically necessary. This was a case-specific intraoperative judgment rather than a predefined protocol. Postpartum anti-D immunoglobulin was not administered, and no postpartum antibody screening was performed. Although the patient was discharged on postoperative day 5 and was clinically well at 3-month follow-up, this interval was too short to evaluate delayed alloantibody formation. Therefore, her subsequent alloimmunization status and the immunohematologic safety of this management could not be determined. This report describes one context-specific decision to prepare and retain ICS blood without subsequent reinfusion. It does not establish that withholding reinfusion is safe or preferable and does not provide a generalizable strategy or threshold for other Rh(D)-negative patients with PAS.

## 1 Introduction

Placenta accreta spectrum (PAS) is among the most hemorrhage-prone conditions in obstetric surgery, and placenta percreta represents its most invasive form. Current PAS guidance emphasizes planned delivery at an experienced center, antenatal optimization of anemia, multidisciplinary surgical and anesthetic planning, early coordination with transfusion services, access to a massive transfusion protocol, and availability of cell-salvage equipment in the operating room (1–3). These measures position intraoperative cell salvage (ICS) as one component of a broader patient blood management strategy rather than as a stand-alone intervention.

Recommendations regarding obstetric ICS remain selective rather than universal. The Association of Anaesthetists does not recommend routine cell salvage for all cesarean deliveries but advises consideration of a “collect-only” approach in women with preoperative anemia, an anticipated high risk of hemorrhage, or unexpected intraoperative bleeding (4). Guidance also varies according to scope and setting: the 2025 World Health Organization guideline for the treatment of postpartum hemorrhage considered the evidence insufficient for routine clinical use and recommended cell salvage only within rigorous research contexts (5). Taken together, current guidance supports selective preparation or availability of ICS in some high-risk obstetric settings while acknowledging continuing uncertainty regarding its routine use. Abdominal aortic balloon occlusion and its combined use with cell salvage have also been described in PAS. Therefore, neither ICS, intermittent abdominal aortic balloon occlusion (IABO), nor their combination constitutes the novel aspect of the present report (6–10).

In Rh(D)-negative women, ICS introduces a specific clinical dilemma. Salvaged blood may still contain fetal red cells despite suction discipline, washing, and leukocyte depletion filtering, and reinfusion may carry residual risk of Rh alloimmunization (11, 12). Current transfusion medicine guidance recommends cord-blood or neonatal RhD typing and postpartum anti-D immunoglobulin for an unsensitized RhD-negative patient who delivers an Rh(D)-positive neonate. Fetomaternal hemorrhage should also be assessed to determine whether additional anti-D dosing is required, and obstetric cell-salvage guidance emphasizes adherence to established anti-D protocols when ICS is used (4, 13, 14). In the Chinese Han population, Rh(D)-negative status occurs in only 0.3%–0.5% of individuals, making compatible allogeneic blood difficult to procure (15). In the Salvage Autologous Blood Versus donor blood in Obstetric haemorrhage (SALVO) trial, the incidence of fetomaternal hemorrhage of ≥2 mL among RhD-negative women delivering Rh(D)-positive neonates was higher in the cell-salvage group than in the control group (25.6% vs 10.5%). However, the trial did not evaluate an RhD-negative PAS subgroup or address whether an already processed ICS unit should be reinfused when hemorrhage has been controlled and additional red-cell support is no longer required (16).

The importance of this case lies not in the rarity of Rh(D)-negative placenta percreta alone, but in illustrating a real-time intraoperative decision point under competing clinical constraints. Procurement of compatible Rh(D)-negative allogeneic blood required advance regional coordination, whereas reinfusion of salvaged blood could add fetal red-cell exposure. During a 4,000-mL hemorrhage, 900 mL of processed autologous blood was maintained as an immediately available contingency. After IABO, definitive surgical hemostasis, and same-type allogeneic transfusion controlled the bleeding and additional red-cell support was no longer considered clinically necessary, the processed blood was not reinfused. The educational message is that the preparation of ICS blood and the decision to reinfuse it are separate steps, and that the need for reinfusion can be reassessed as hemorrhage control and transfusion requirements evolve. This single case illustrates a patient-specific decision process and does not establish a generalizable management strategy or threshold for withholding reinfusion.

## 2 Case description

### 2.1 Patient information

A 29-year-old woman (gravida 3, para 2) was admitted at 30 weeks of gestation for inpatient surveillance because of progressively concerning features of placenta accreta and recurrent small-volume antepartum bleeding. She had two previous cesarean deliveries, both reportedly resulting in Rh(D)-positive neonates, but had not received anti-D immunoglobulin after either delivery. Indirect antiglobulin testing was negative on three separate antenatal samples. Preoperative diagnoses included complete placenta previa, placenta percreta, scarred uterus, intrahepatic cholestasis of pregnancy, moderate anemia, Rh(D)-negative blood type, and subclinical hypothyroidism. Extended Rh phenotyping showed RhC positivity and RhE negativity. RHD genotyping for DEL detection was not performed because molecular blood-group testing was not available within the inpatient timeframe at our institution. For future similar high-risk cases, a feasible institutional pathway would involve identifying the need for RHD genotyping during antenatal admission or early multidisciplinary planning, consulting transfusion medicine at that stage, and sending a pretransfusion blood sample to a qualified regional blood center or molecular immunohematology reference laboratory. Such a pathway would require predefined specimen requirements, transport arrangements, expected turnaround times, and procedures for incorporating the result into the transfusion record and electronic medical record. Until a validated genotype result is available, the patient should continue to be managed conservatively as serologically Rh(D)-negative.

The decision to deliver at 32 weeks of gestation was reached by multidisciplinary consensus after balancing several factors: recurrent small-volume antepartum bleeding during admission; persistent anemia despite iron supplementation (hemoglobin trended from 83 g/L on admission to 79 g/L on hospital day 7 before partial recovery to 86 g/L the day before surgery); worsening intrahepatic cholestasis of pregnancy; and the perceived risk of unscheduled catastrophic hemorrhage at later gestation. Antenatal corticosteroids and magnesium sulfate for fetal neuroprotection were administered before delivery.

### 2.2 Clinical findings and diagnostic assessment

Baseline laboratory evaluation 1 day before surgery showed hemoglobin (Hb) 86 g/L, red blood cell (RBC) count 3.07   ×   10^12^/L, and platelet (PLT) count 237   ×   10⁹/L. Coagulation parameters were within normal limits (prothrombin time [PT] 10.7 s, international normalized ratio [INR] 0.92, activated partial thromboplastin time [APTT] 29.7 s, thrombin time [TT] 15.7 s, fibrinogen 4.30 g/L). Serum creatinine was 34 µmol/L and albumin 34.0 g/L (Tables 1–3). Hematology results from January 17 (admission) and January 24 (routine follow-up) provide longitudinal context (Table 1).

**Table 1:** Serial hematology parameters. Units: RBC   ×  10^12^/L; Hb g/L; WBC   ×  10⁹/L; Neut   ×  10⁹/L; PLT   ×  10⁹/L.

Date	Time	RBC	Hb	WBC	Neut	PLT
Jan 17	—	2.90	83	10.50	8.45	208
Jan 24	—	2.88	79	10.10	7.48	262
Jan 27	—	3.07	86	9.71	7.09	237
Jan 28	10:50	2.79	80	10.58	8.24	196
Jan 28	11:21	2.88	86	11.56	9.24	168
Jan 28	12:45	2.95	87	21.60	20.30	137
Jan 28	16:13	3.38	99	21.76	20.81	161
Jan 28	20:26	3.43	100	26.72	25.40	254
Jan 29	—	3.16	95	23.27	20.75	153
Jan 31	—	3.15	91	14.59	11.46	161
Feb 2	—	3.45	100	10.67	8.31	236

**Table 2:** Serial coagulation parameters. Units: PT s; INR; TT s; APTT s; Fbg g/L; D-dimer µg/mL.

Date	Time	PT	INR	TT	APTT	Fbg	D-dimer
Jan 17	—	10.2	0.88	16.2	27.1	3.75	—
Jan 24	—	10.7	0.92	15.7	29.7	4.30	—
Jan 28	11:31	11.4	0.99	79.1	71.4	3.75	1.7
Jan 28	12:13	11.9	1.04	18.5	51.0	2.15	1.1
Jan 28	12:58	11.2	0.97	18.0	42.3	2.51	1.2
Jan 28	16:12	10.7	0.92	17.0	34.7	2.96	1.4
Jan 29	12:05	10.2	0.88	14.5	33.5	4.64	2.5
Jan 31	10:53	10.6	0.92	15.2	32.5	5.18	1.6

**Table 3:** Thromboelastography results. Units: CI dimensionless; R min; K min; angle°; MA mm; LY30%.

**Date**	**Time**	**CI**	**R**	**K**	**Angle**	**MA**	**LY30**
Jan 28	12:21	0.0	5.2	1.8	67.6	54.8	0
Jan 28	16:08	−0.1	5.0	1.9	66.9	53.5	0

Preoperative magnetic resonance imaging (MRI) demonstrated complete placenta previa involving the lower uterine segment at the prior cesarean scar, with loss of the normal uterine–bladder interface and features concerning for bladder invasion (Figure 1).

**Figure 1:**
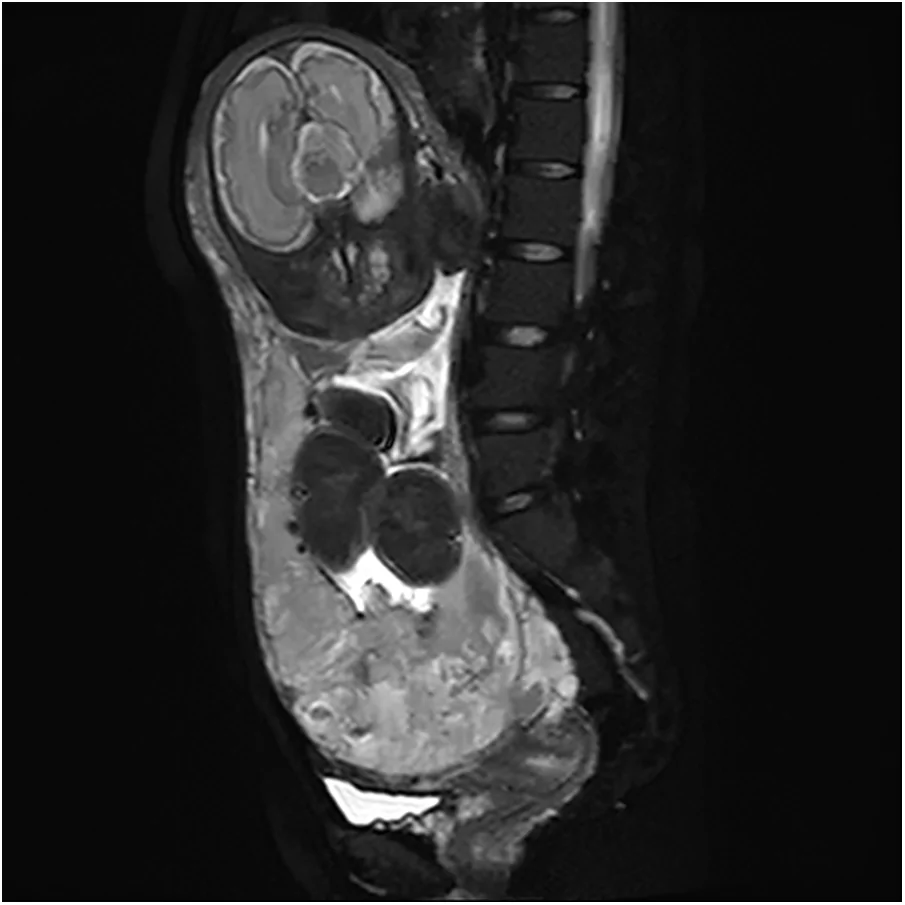
Sagittal preoperative pelvic magnetic resonance imaging demonstrating complete placenta previa, marked thinning of the lower uterine segment at the previous cesarean scar, loss of the normal uterine–bladder interface, and findings raising clinical suspicion of placenta percreta with possible bladder involvement.

### 2.3 Timeline

A perioperative timeline showing preparation, hemorrhage-control measures, transfusion, ICS management, laboratory monitoring, and recovery is shown in Figure 2. Serial hematology, coagulation, and viscoelastic results are provided in Tables 1–3.

**Figure 2:**
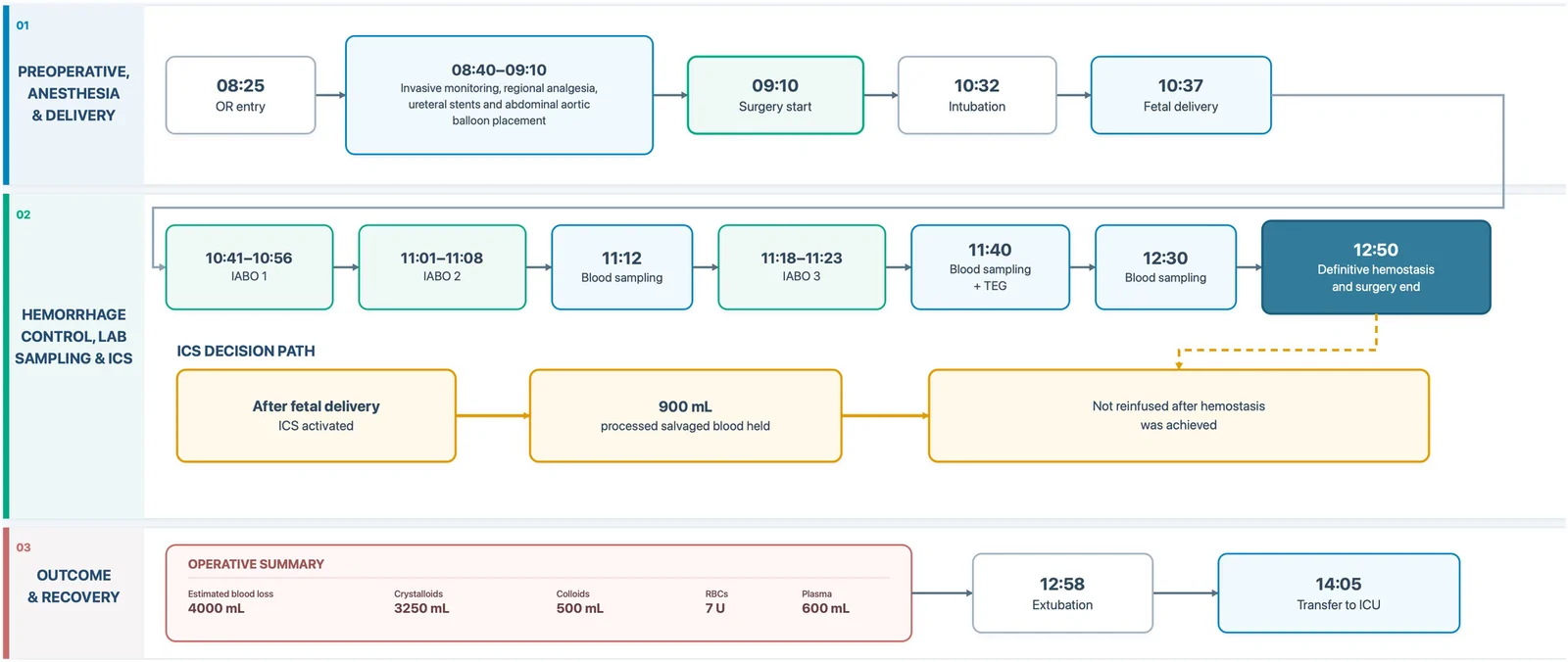
Perioperative timeline. The three horizontal sections summarize (1) preoperative preparation, anesthesia, and fetal delivery; (2) hemorrhage control, blood sampling, and the intraoperative cell salvage (ICS) decision pathway; and (3) operative outcomes and postoperative recovery. Three periods of intermittent abdominal aortic balloon occlusion (IABO) were used during hemorrhage management. Times accompanying the blood-sampling nodes (11:12, 11:40, and 12:30) indicate sample-collection times; the corresponding result-reporting times and laboratory values are presented in Tables 1–3. The timeline is schematic and not drawn to scale. IABO, intermittent abdominal aortic balloon occlusion; ICS, intraoperative cell salvage; ICU, intensive care unit; TEG, thromboelastography.

### 2.4 Multidisciplinary planning and shared decision-making

A multidisciplinary team comprising obstetrics, anesthesiology, vascular intervention, urology, transfusion medicine, intensive care, and neonatology convened twice before surgery. Planning specifically addressed: (i) the high anticipated blood loss; (ii) the constrained allogeneic supply for an O Rh(D)-negative patient — 4 U of cross-matched O Rh(D)-negative packed red blood cells (PRBC) and 600 mL of fresh frozen plasma (FFP) were sequestered and verified in the operating room before incision, with an additional same-type reserve mobilized from the regional blood bank; (iii) the role of IABO as the primary mechanical hemostatic adjunct; and (iv) ICS as a contingency for refractory hemorrhage if allogeneic supply became insufficient.

Preoperative autologous blood donation was not pursued because (a) maternal anemia (Hb 79–86 g/L) precluded further phlebotomy without compromising oxygen delivery; (b) phlebotomy mobilization risked precipitating further antepartum bleeding; and (c) the preoperative interval was insufficient for adequate erythropoietic recovery between donations.

Shared decision-making with the patient was conducted in two dedicated consultations attended by the patient, her partner, the obstetric and anesthesia teams, and a transfusion medicine specialist. The discussions covered: (i) anticipated blood loss and the likelihood of cesarean hysterectomy; (ii) the limited compatible-blood supply and the rationale for ICS preparation; (iii) the residual immunohematologic risk associated with ICS reinfusion in Rh(D)-negative women; (iv) the use of IABO and ureteral stents; (v) the alternative of regional anesthesia and reasons general anesthesia was preferred for hemorrhage scenarios; and (vi) postoperative ICU care. The patient understood that ICS would be prepared as a contingency and that reinfusion would depend on the intraoperative clinical need for additional red-cell support. Written clinical consent was obtained for anesthesia, the surgical procedure, ICS preparation and conditional reinfusion, IABO, and ureteral stenting. Separately, written informed consent was obtained from the patient for publication of this case report and any potentially identifiable images or data.

### 2.5 Therapeutic intervention

#### 2.5.1 Anesthetic and interventional preparation

A left radial arterial catheter and right internal jugular venous catheter were placed for invasive monitoring. Bilateral transversus abdominis plane and rectus sheath blocks were performed for multimodal analgesia. Bilateral ureteral stents were placed before induction of general anesthesia, and an abdominal aortic balloon catheter was inserted via the right femoral artery. Cesarean delivery followed by subtotal hysterectomy was performed under general anesthesia with lung-protective ventilation.

#### 2.5.2 Surgical procedure and intraoperative course

At laparotomy, dense pelvic and bowel adhesions required adhesiolysis. Intraoperative findings confirmed full-thickness myometrial invasion at the prior cesarean scar with extension toward the bladder wall. The placenta could not be safely separated. A live male neonate was delivered at 10:37 (2,400 g; Apgar 5/9/10) and transferred to the NICU for prematurity, where ABO/RhD typing on day 1 confirmed Rh(D)-positive status; short-term outcome was favorable.

Because placental separation was not feasible, cesarean subtotal hysterectomy was performed for definitive hemorrhage control. Surgery was completed at 12:50 (operative time 3 h 40 min). Estimated blood loss was 4,000 mL, with peak hemorrhage between 10:40 and 11:30 during placental manipulation and uterine devascularization.

Intermittent abdominal aortic balloon occlusion was used during the most hemorrhage-prone phases (three inflations of 15, 7, and 5 min with 5–10-minute reperfusion intervals between inflations) (6, 7, 10). Tranexamic acid 0.5 g was administered at 11:05.

Intraoperative fluid therapy comprised 3,250 mL of crystalloid and 500 mL of colloid. Transfusion was initiated at the onset of peak hemorrhage at 10:40, and the point-of-care Hb measured at 10:50 was 80 g/L. Allogeneic transfusion consisted of seven units of cross-matched O Rh(D)-negative PRBCs, sequentially released from the operating-room reserve and regional blood bank and administered through a level-1 rapid infusor with an in-line fluid warmer, together with 600 mL of FFP. Coagulation support was guided by serial clinical and laboratory assessment. Cumulative transfusion timing and intraoperative laboratory values are shown in Figure 2 and Tables 1–3.

#### 2.5.3 Intraoperative cell salvage: preparation, processing, and reserve decision

ICS was prepared in advance and activated only after fetal delivery (10:37) and clearance of amniotic fluid through suction discipline. The first salvage processing event was documented at 10:40, coinciding with peak hemorrhage. A total of 900 mL of processed autologous blood was held in a separate reservoir, with a leukocyte depletion filter staged for use in the event of reinfusion. ICS was therefore physically available throughout the high-risk window of 10:40–11:30.

The decision not to reinfuse the processed blood was made intraoperatively after surgical hemostasis had been achieved. The following factors were subsequently summarized from the clinical course as the case-specific considerations underlying this decision:

1. Hemorrhage during the peak window was controlled mechanically and surgically. IABO and uterine devascularization, supported by parallel transfusion of cross-matched O Rh(D)-negative PRBC and FFP, restored hemodynamic stability. Definitive hemostasis was achieved via cesarean subtotal hysterectomy.
2. No further red-cell support was considered clinically necessary. Hemoglobin nadired at 80 g/L at 10:50, was maintained between 86 and 87 g/L during ongoing replacement (Table 1), and the patient remained hemodynamically stable on minimal vasopressor support after definitive hemostasis.
3. The residual immunohematologic risk was uncertain. Washing and planned leukocyte-depletion filtration could reduce but not eliminate fetal red-cell exposure; however, contamination of the processed blood was not measured in this case. Once no further red-cell support was considered clinically necessary, reinfusion offered no evident immediate clinical benefit.

The decision to withhold reinfusion was therefore based on resolution of hemorrhage and adequate oxygen delivery rather than on viscoelastic testing. Salvaged, washed red cells contain almost no clotting factors, and thromboelastography (TEG) reflects coagulation function rather than oxygen-carrying capacity. The TEG result obtained at 12:21 (after surgical hemostasis was achieved) instead informed the decision not to escalate factor- and platelet-based hemostatic therapy and supported continued observation of an evolving but stabilizing coagulation profile (17, 18). Maternal stabilization at the close of surgery resulted from timely IABO, definitive surgical hemostasis, and same-type allogeneic transfusion administered during the active hemorrhage window; ICS functioned as a prepared but unused contingency.

#### 2.5.4 Coagulation and laboratory trajectory

Serial laboratory monitoring documented changes during hemorrhage and resuscitation. Hb decreased from 86 g/L preoperatively to a nadir of 80 g/L at 10:50, increased to 86 g/L at 11:21 and 87 g/L at 12:45 during concurrent red-cell replacement, and was 100 g/L at discharge on postoperative day 5 (Table 1). Conventional coagulation assays showed transient abnormalities at 11:31, including markedly prolonged APTT and TT. TEG performed at 12:21 did not indicate marked global hypocoagulability [R 5.2 min; maximum amplitude (MA) 54.8 mm; LY30 0%] (Tables 2 and 3) (Figure 3). The dissociation between conventional and viscoelastic results contributed to the decision not to escalate hemostatic therapy further at that stage. Albumin decreased from 34.0 to 23.2 g/L intraoperatively with partial recovery thereafter, while renal function remained preserved.

**Figure 3:**
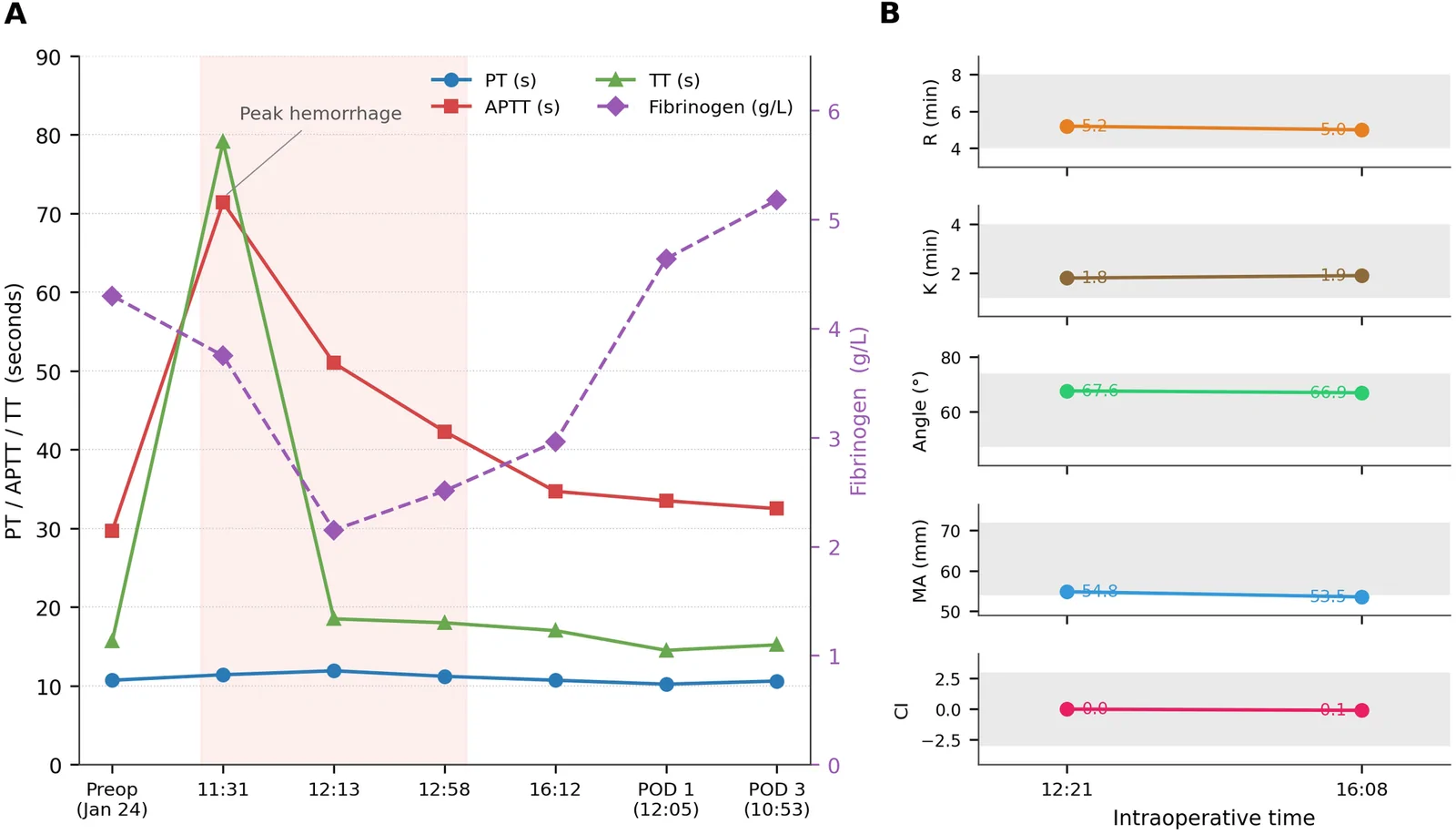
Dynamic perioperative coagulation assessment. **(A)** Serial conventional coagulation measurements plotted according to result-reporting time; the shaded area indicates the period of peak hemorrhage. **(B)** Thromboelastography measurements reported at 12:21 and 16:08. PT, prothrombin time; APTT, activated partial thromboplastin time; TT, thrombin time; R, reaction time; K, clot-formation time; MA, maximum amplitude; CI, coagulation index.

### 2.6 Follow-up and outcomes

Mechanical ventilation was discontinued at 12:55 and extubation completed at 12:58 — 8 min after the conclusion of surgery. Postoperative analgesia was patient-controlled intravenous tramadol 1 g and dexamethasone 15 mg in 150 mL at 2.5 mL/h. The patient was transferred to the ICU at 14:05, observed for 1 day, and stepped down to the general ward. No additional transfusion was required postoperatively, and no major surgical, thromboembolic, or clinically apparent acute transfusion-related complications occurred.

Postpartum anti-D immunoglobulin was not administered. No contemporaneous transfusion medicine or multidisciplinary team documentation of the rationale for this decision was available. Fetomaternal hemorrhage was not quantified using either a Kleihauer–Betke acid-elution test or flow cytometry, and postoperative antibody screening was not performed. Therefore, the subsequent maternal alloimmunization status could not be determined. Although hysterectomy eliminated the risk to a future pregnancy, alloimmunization could remain relevant to future transfusion support. The non-administration of anti-D is therefore reported as an incompletely documented management decision and should not be interpreted as a recommended approach. In future similar cases, an unsensitized, serologically Rh(D)-negative patient who delivers an Rh(D)-positive neonate should receive postpartum anti-D immunoglobulin as soon as possible and within 72 h of delivery, regardless of future fertility. A postpartum maternal sample should be obtained to quantify fetomaternal hemorrhage using a validated method, such as the Kleihauer–Betke test or flow cytometry. The standard postpartum dose specified by the applicable local or national protocol should be administered without waiting for the fetomaternal hemorrhage result. If the quantified hemorrhage exceeds the volume covered by the standard dose, additional anti-D should be calculated and administered promptly in consultation with the transfusion service (13, 14).

Histopathologic examination of the hysterectomy specimen demonstrated focal myometrial thinning with placental villi extending through the full thickness of the myometrium, consistent with placenta accreta spectrum; no chorionic vascular malformation was identified. Bilateral ureteral stents were removed before discharge without complication. The patient was discharged on postoperative day 5 with Hb 100 g/L.

At 3-month outpatient follow-up the patient was clinically well, with no wound, urinary, or thromboembolic complications, and had returned to normal daily activity. When asked about her perioperative experience as part of routine follow-up, she reported that her main preoperative concerns had been the rarity of her blood type and the possibility of needing massive transfusion, and that the preoperative multidisciplinary consultations had clarified for her the rationale for ICS preparation and IABO and that the use of the salvaged blood would depend on the intraoperative clinical requirement for additional red-cell support. She expressed no regret about the decision not to reinfuse the salvaged unit once she was informed that hemorrhage had been controlled by other means.

The 3-month follow-up was limited to assessment of clinical recovery and did not include a maternal red-cell antibody screen. This duration was too short to exclude delayed alloantibody formation. In future similar cases, immunohematologic follow-up should continue for a minimum of 6 months after delivery and should include a maternal red-cell antibody screen at approximately 6 months postpartum. If the screening result is positive, the antibody specificity should be identified and permanently documented in the patient's transfusion record to inform future transfusion support.

## 3 Discussion

This case illustrates the distinction between preparing ICS for anticipated catastrophic hemorrhage and deciding whether the processed blood remains clinically necessary after hemorrhage control. The favorable maternal outcome should be attributed to the combined hemorrhage-management strategy rather than to ICS alone. During the period of peak bleeding, IABO, consisting of three inflation periods of 15, 7, and 5 min, was intended to provide temporary proximal flow control and may have facilitated surgical exposure and operative progression. However, its independent effect on blood loss cannot be quantified from this single case. Definitive source control was achieved through surgical devascularization and subtotal hysterectomy, while seven units of cross-matched O Rh(D)-negative packed red blood cells and 600 mL of fresh frozen plasma provided oxygen-carrying capacity and hemostatic support. By contrast, the 900 mL of processed autologous blood was not reinfused and therefore did not directly contribute to hemodynamic stabilization. Its role was to provide an additional reserve in case hemorrhage continued or compatible allogeneic blood became insufficient.

The evidence supporting the individual components of this approach differs in strength. In the randomized SALVO trial, routine cell salvage during cesarean delivery did not significantly reduce the overall rate of donor blood transfusion, although the adjusted analysis suggested a possible treatment effect (16). The trial was not designed specifically for Rh(D)-negative patients with PAS, nor did it evaluate whether a processed ICS unit should be reinfused after surgical hemostasis had already been achieved. Observational studies of ICS and IABO in PAS have reported their feasibility and potential associations with reduced allogeneic transfusion or blood loss; however, these findings are susceptible to patient selection, procedural heterogeneity, and confounding (6–10). Professional recommendations, which are largely based on observational evidence and expert consensus, emphasize multidisciplinary preparation, availability of compatible blood components, and selective or collection-only use of ICS in patients at high risk of obstetric hemorrhage (2–5). The guidance considered here does not provide a validated threshold for withholding or proceeding with reinfusion of a processed ICS unit. Therefore, the decision in this case represents individual intraoperative clinical judgment rather than the application of a trial-derived recommendation, published reinfusion threshold, or institutional protocol.

The potential immunohematologic risk of ICS must be balanced against the immediate consequences of uncontrolled hemorrhage. Historical estimates suggest that, without anti-D prophylaxis, Rh(D) alloimmunization occurs in approximately 13%–16% of susceptible Rh(D)-negative women after delivery of an Rh(D)-positive infant. Postpartum prophylaxis reduces this risk to approximately 0.5%–1.8%, and combined antepartum and postpartum prophylaxis reduces it further to approximately 0.14%–0.2% (14). These estimates describe exposure associated with pregnancy and delivery and should not be interpreted as the incremental risk attributable specifically to ICS. In the SALVO trial, among Rh(D)-negative women who delivered Rh(D)-positive infants, fetomaternal hemorrhage of at least 2 mL was detected more frequently in the cell-salvage group than in the control group (25.6% vs 10.5%; adjusted odds ratio, 5.63; 95% confidence interval, 1.43–22.14) (16). However, the trial did not assess subsequent maternal alloimmunization; therefore, this laboratory finding cannot establish the long-term immunologic risk associated with ICS reinfusion.

In contrast, ongoing PAS-related hemorrhage presents an immediate and potentially fatal threat. If active bleeding, hemodynamic instability, inadequate oxygen delivery, or insufficient compatible allogeneic blood had persisted, the clinical benefit of reinfusing the salvaged blood would likely have outweighed the uncertain incremental immunohematologic risk. In this patient, by the time reinfusion was considered, the bleeding source had been surgically controlled following temporary IABO support, the hemoglobin concentration remained at or above 80 g/L, hemodynamic stability had been restored, and no additional red-cell support was considered clinically necessary. The control of hemorrhage, absence of a further red-cell transfusion requirement, and uncertain immunohematologic risk were retrospectively summarized as the case-specific considerations underlying non-reinfusion. They were not predefined criteria, were not agreed upon during preoperative multidisciplinary planning, and were not derived from published recommendations or institutional guidance. Accordingly, these considerations should not be interpreted as a generalizable ICS reinfusion threshold. Importantly, non-reinfusion was considered only after the immediate indication for additional red-cell support had resolved.

The postpartum immunohematologic management also requires cautious interpretation. The neonate was Rh(D)-positive, but anti-D immunoglobulin was not administered. There was no contemporaneous transfusion medicine or multidisciplinary team record documenting the rationale for this decision; therefore, the omission of anti-D cannot be presented as a formal protocol-based management decision. Current guidance generally recommends postpartum anti-D prophylaxis for a nonsensitized Rh(D)-negative patient after delivery of an Rh(D)-positive infant, together with assessment of fetomaternal hemorrhage to determine whether additional dosing is required (4, 13, 14). Although hysterectomy eliminates the risk to a future pregnancy, maternal alloimmunization may remain relevant to future transfusion support. Fetomaternal hemorrhage was not quantified in this patient, and postoperative antibody screening was not performed. Consequently, the occurrence or absence of subsequent alloimmunization cannot be determined, and the non-administration of anti-D should not be interpreted as a recommended component of this case-management approach. Future similar cases should follow established postpartum prophylaxis: anti-D immunoglobulin should be administered as soon as possible and within 72 h after delivery of an Rh(D)-positive neonate, irrespective of future fertility. Fetomaternal hemorrhage should be quantified using a validated Kleihauer–Betke acid-elution test or, where available, flow cytometry, and additional anti-D should be administered when the measured hemorrhage exceeds the coverage of the standard dose specified by the applicable protocol (13, 14). Furthermore, the clinically reassuring 3-month follow-up in this case should not be interpreted as evidence that alloimmunization did not occur, because the follow-up duration was short and no maternal antibody screening was performed. Future similar cases should include immunohematologic follow-up for a minimum of 6 months, with a maternal red-cell antibody screen performed at approximately 6 months postpartum and antibody identification undertaken if the screening result is positive (19).

RHD genotyping was not performed in this patient. The negative antenatal antibody screens and previous obstetric history do not establish the presence of a DEL phenotype, and the hypothetical possibility of DEL did not inform the intraoperative ICS or postpartum anti-D decisions. DEL should therefore not be used retrospectively to explain the absence of previously detected alloimmunization or to suggest a lower risk associated with ICS reinfusion. For future similar high-risk cases, access to RHD genotyping could be improved through an institutional referral pathway initiated during antenatal admission or early multidisciplinary planning. This pathway should include early consultation with transfusion medicine, collection and referral of a pretransfusion sample to a qualified regional blood center or molecular immunohematology reference laboratory, use of a validated assay capable of identifying clinically relevant variants including Asian-type DEL (RHD*DEL1), predefined specimen-transport and turnaround arrangements, and permanent documentation of the result in the transfusion record. Until a validated result is available, the patient should be managed conservatively as serologically Rh(D)-negative. Although such testing may improve preoperative immunohematologic characterization, its influence on ICS reinfusion decisions requires further investigation (15).

The perioperative coagulation results require interpretation in relation to their timing and the different analytical characteristics of conventional and viscoelastic testing. At 11:31, the activated partial thromboplastin time and thrombin time were markedly prolonged to 71.4 and 79.1 s, respectively, whereas the prothrombin time was 11.4 s, the international normalized ratio was 0.99, and the fibrinogen concentration remained 3.75 g/L. This sample was obtained from venous blood, and the patient had not been exposed to heparin. The combination of markedly prolonged activated partial thromboplastin time and thrombin time with a near-normal prothrombin time/international normalized ratio and preserved fibrinogen was not typical of generalized dilutional or consumptive coagulopathy. However, because no mixing study, anti-Xa assay, or other targeted investigation was performed, the cause of this transient abnormality cannot be determined retrospectively, and a preanalytical or transient assay-related effect cannot be excluded.

The conventional coagulation results subsequently improved. At 12:13, the activated partial thromboplastin time remained prolonged at 51.0 s, whereas the thrombin time had decreased to 18.5 s and the fibrinogen concentration was 2.15 g/L. At 12:21, thromboelastography (TEG) showed a reaction time of 5.2 min, a clot-formation time of 1.8 min, an *α*-angle of 67.6°, a maximum amplitude of 54.8 mm, a coagulation index of 0.0, and no clot lysis at 30 min; similar findings were obtained at 16:08. These parameters did not indicate marked impairment of global clot initiation, propagation, strength, or fibrinolysis at those time points. The apparent discrepancy does not necessarily represent a contradiction because conventional assays evaluate selected coagulation endpoints in platelet-poor plasma, whereas TEG assesses dynamic clot formation in whole blood and incorporates the contributions of coagulation factors, fibrinogen, platelets, and fibrinolysis (17, 18). The TEG findings therefore did not invalidate the prolonged conventional coagulation results. When interpreted together with achieved surgical hemostasis and clinical stability, they did not indicate a need for further escalation of factor or platelet replacement. TEG was not used independently to determine the requirement for red-cell transfusion or ICS reinfusion.

Overall, this case illustrates that preparation for anticipated catastrophic hemorrhage and the subsequent use of a prepared blood-conservation resource are related but distinct decisions. ICS provided contingency capacity within a broader strategy centered on temporary flow control, definitive surgical hemostasis, and compatible allogeneic transfusion. This single case does not establish that withholding ICS reinfusion is safer or more effective in Rh(D)-negative patients with PAS, nor does it support a fixed reinfusion threshold or management strategy. Rather, it generates the hypothesis that the reinfusion decision may be reassessed dynamically according to ongoing blood loss, hemodynamic and oxygen-delivery requirements, the availability of compatible allogeneic blood, and relevant immunohematologic considerations.

### 3.1 Strengths and limitations

Strengths of this report include the detailed description of the intraoperative decision-making process regarding ICS reinfusion, the serial laboratory and viscoelastic data, the integration of the patient perspective into 3-month follow-up, and the transparent reporting of incompletely documented or uncertain management decisions.

The principal limitations arise from the single-case design and incomplete immunohematologic evaluation. The salvaged blood was not reinfused, and this report has no comparator. Consequently, this case cannot evaluate the safety of ICS reinfusion, compare outcomes between reinfusion and non-reinfusion, or establish that withholding reinfusion was safe or effective. The favorable maternal outcome followed IABO, definitive surgical hemostasis, and compatible allogeneic transfusion and therefore cannot be attributed to non-reinfusion of the salvaged blood. Fetal red-cell contamination of the processed blood was not measured. RHD genotyping was not performed, postpartum anti-D immunoglobulin was not administered, and no contemporaneous transfusion medicine or multidisciplinary team record documented the rationale for this decision. Fetomaternal hemorrhage was not quantified, and postoperative antibody screening was not performed; therefore, the occurrence or absence of subsequent maternal alloimmunization cannot be determined. These constraints define the boundaries of inference from this single case. In addition, the 3-month follow-up was limited to clinical recovery and was too short to evaluate delayed alloantibody formation; no maternal red-cell antibody screen was obtained during follow-up.

## 4 Conclusion

This case describes individualized perioperative reassessment of ICS use in an Rh(D)-negative patient with placenta percreta. Maternal stabilization was achieved through IABO, definitive surgical hemostasis, and compatible allogeneic transfusion; the 900 mL of processed autologous blood remained an unused reserve and did not directly contribute to stabilization. In this single well-managed patient, ICS preparation ensured that autologous red cells were immediately available during major hemorrhage, but this observation does not establish the safety, efficacy, or generalizability of preparing ICS without subsequent reinfusion or of withholding reinfusion after hemorrhage control. Larger prospective studies are required to determine whether such preparation improves clinical preparedness or outcomes and to clarify the circumstances in which processed ICS blood should be reinfused.

## Data Availability

The original contributions presented in the study are included in the article/Supplementary Material, further inquiries can be directed to the corresponding author.
