# Supplementary material for: Individualized perioperative decision-making for intraoperative cell salvage in an Rh(D)-negative patient with placenta percreta: a case report
Source: Front Med (Lausanne). 2026 Sep 30;13:1874079. doi: 10.3389/fmed.2026.1874079 (PMC13661923; doi:10.3389/fmed.2026.1874079)
Supplement: Supplementary file 1 [file Datasheet1.pdf]

CARE Checklist for Case Reports

Supplementary Material — Completed CARE Checklist (2013)

Manuscript title: Individualized perioperative decision-making for intraoperative cell salvage in an Rh(D)-negative patient with placenta percreta: a case report

Authors: Yanping Lu, Xuewei Wang, Cuiyuan Huang, Henjing Zou, Changhui Shao, Shan Ou

Journal: Frontiers in Global Women’s Health (Section: Maternal Health)

Please indicate in which section each item has been reported in your manuscript. The checklist follows the CARE (CAse REport) guidelines (Riley et al., J Clin Epidemiol, 2017).

Topic	Item	Checklist Item Description	Reported on	Location in Manuscript
**Title**	1	The diagnosis or intervention of primary focus followed by the words "case report".	Title page	Title identifies individualized perioperative decision-making for intraoperative cell salvage in an Rh(D)-negative patient with placenta percreta and ends with "a case report".
**Keywords**	2	2 to 5 key words that identify diagnoses or interventions in this case report, including "case report".	Title page (Keywords)	Keywords include intraoperative cell salvage; placenta accreta spectrum; placenta percreta; Rh(D)-negative; IABO; obstetric anesthesia; patient blood management; case report.
**Abstract**	3a	Introduction: What is unique about this case and what does it add to the scientific literature?	Abstract	Abstract describes the context-specific dilemma of preparing ICS for anticipated catastrophic hemorrhage and then reassessing whether the processed blood remains clinically necessary after hemorrhage control in an Rh(D)-negative patient.
	3b	The patient’s main concerns and important clinical findings.	Abstract	Abstract summarizes the patient’s gestational status, Rh(D)-negative blood type, anemia, placenta percreta, and suspected bladder invasion.
	3c	The primary diagnoses, interventions, and outcomes.	Abstract	Abstract reports placenta percreta with suspected bladder invasion, IABO, tranexamic acid, same-type allogeneic transfusion, ICS preparation

				without reinfusion, cesarean subtotal hysterectomy, clinical recovery, non-administration of postpartum anti-D, and no postoperative antibody screening.
	3d	Conclusion: What are the "take-away" lessons?	Abstract	Abstract conclusion emphasizes that this was a single context-specific decision to prepare and retain ICS blood without reinfusion and does not establish that withholding reinfusion is safe, preferable, or a generalizable threshold for other Rh(D)-negative patients with PAS.
**Introduction**	4	Briefly summarize why this case is unique with medical literature references.	Section 1 Introduction	Introduction explains current PAS and obstetric ICS guidance, the residual fetal red-cell and alloimmunization concern in Rh(D)-negative patients, constrained compatible allogeneic supply, and the evidence gap regarding whether processed ICS blood should be reinfused after hemorrhage control; cites refs 1–16.
**Patient Information**	5a	De-identified demographic and other patient-specific information.	Section 2.1 Patient Information	De-identified demographics: 29-year-old gravida 3, para 2 woman at 32 weeks of gestation with O Rh(D)-negative blood type.
	5b	Primary concerns and symptoms of the patient.	Section 2.1 Patient Information	Primary concerns were planned delivery for placenta percreta with suspected bladder invasion, anticipated major hemorrhage, and constrained allogeneic supply due to Rh(D)-negative status.
	5c	Medical, family, and psychosocial history including relevant genetic information.	Section 2.1 Patient Information	Relevant history includes two prior cesarean deliveries with reportedly Rh(D)-positive neonates and no anti-D prophylaxis; recurrent small-volume antepartum bleeding during admission; intrahepatic cholestasis of pregnancy; and subclinical hypothyroidism. RHD genotyping for DEL detection was not performed because molecular testing was unavailable within the inpatient timeframe; the manuscript outlines an antenatal referral pathway for future similar

				cases.
	5d	Relevant past interventions with outcomes.	Section 2.1 Patient Information	Two previous cesarean deliveries with Rh(D)-positive neonates; no prior anti-D immunoglobulin prophylaxis was administered.
**Clinical Findings**	6	Describe significant physical examination (PE) and important clinical findings.	Section 2.2 Clinical Findings; Tables 1–3	Important clinical findings include preoperative anemia (Hb 86 g/L), MRI features concerning for bladder invasion, 4,000 mL intraoperative blood loss with peak hemorrhage 10:40–11:30, transient intraoperative coagulopathy, and serial laboratory/viscoelastic data.
**Timeline**	7	Historical and current information from this episode of care organized as a timeline.	Section 2.3 Timeline; Figure 2; Table 1	Figure 2 provides the perioperative timeline (preparation, IABO inflations, transfusion, ICS, postoperative recovery); Table 1 summarizes the chronological hematologic course.
**Diagnostic Assessment**	8a	Diagnostic methods (PE, laboratory testing, imaging, surveys).	Section 2.2; Tables 1–3	Diagnostic methods included blood typing, extended Rh phenotyping, indirect antiglobulin testing, MRI, serial hematology and coagulation testing, and thromboelastography.
	8b	Diagnostic challenges.	Sections 2.1, 3.1; Limitations	RHD genotyping and fetal red-cell contamination of the processed blood were not assessed; fetomaternal hemorrhage was not quantified by Kleihauer–Betke testing or flow cytometry; no postoperative or follow-up maternal antibody screen was performed; and 3-month clinical follow-up was too short to exclude delayed alloantibodies.
	8c	Diagnosis (including other diagnoses considered).	Section 2.1; Section 2.6 (histology)	Primary diagnoses were complete placenta previa and placenta percreta with suspected bladder invasion in an Rh(D)-negative woman; placenta accreta spectrum was confirmed histopathologically (subtotal hysterectomy specimen).
	8d	Prognostic characteristics when applicable.	N/A	Not applicable — this is a perioperative case report rather than a report centered on prognostic

				staging or long-term prognostic classification.
**Therapeutic Intervention**	9a	Types of intervention (pharmacologic, surgical, preventive).	Sections 2.5.1–2.5.3	Interventions included general anesthesia with invasive monitoring, regional analgesic blocks, bilateral ureteral stents, IABO, tranexamic acid, allogeneic PRBC and FFP transfusion, ICS preparation, and cesarean subtotal hysterectomy.
	9b	Administration of intervention (dosage, strength, duration).	Sections 2.5.2–2.5.3	Administration details: tranexamic acid 0.5 g; three IABO inflations of 15, 7, and 5 minutes with 5–10-minute reperfusion intervals; 7 U of cross-matched O Rh(D)-negative PRBC; 600 mL of FFP; 900 mL of processed salvaged blood (held in reserve, not reinfused).
	9c	Changes in therapeutic interventions with explanations.	Section 2.5.3	After hemostasis, non-reinfusion was retrospectively attributed to case-specific considerations: bleeding was controlled, no further red-cell support was needed, and immunohematologic risk remained uncertain. These were not predefined criteria, a protocol, or a generalizable threshold. TEG guided hemostatic therapy, not the red-cell or ICS decision.
**Follow-up and Outcomes**	10a	Clinician- and patient-assessed outcomes when available.	Section 2.6 Follow-up and Outcomes	No major surgical, thromboembolic, or clinically apparent acute transfusion-related complications occurred; ICU observation lasted 1 day; discharge was on postoperative day 5 with Hb 100 g/L. At 3 months the patient was clinically well, had resumed normal activity, and expressed no regret about non-reinfusion. Follow-up assessed clinical recovery only and was too short to evaluate delayed alloantibodies.
	10b	Important follow-up diagnostic and other test results.	Section 2.6; Tables 1–3	Follow-up data include serial hematology, coagulation, and TEG results; histopathologic confirmation of PAS; and neonatal RhD-positive typing on day 1. Fetomaternal hemorrhage was not quantified, and no postoperative or 3-month

				maternal antibody screen was performed.
	10c	Intervention adherence and tolerability.	Section 2.6	All perioperative interventions were tolerated without documented intolerance; no additional transfusion was required postoperatively.
	10d	Adverse and unanticipated events.	Section 2.5.2; Section 2.6	Major hemorrhage with transient intraoperative coagulopathy occurred (estimated blood loss 4,000 mL); no major postoperative complications were recorded.
**Discussion**	11a	Strengths and limitations in your approach to this case.	Section 3.1 Strengths and Limitations	Strengths: detailed ICS decision chronology, serial laboratory/TEG data, patient perspective, and transparent reporting of uncertainty. Limitations: single case without a comparator; salvaged blood not reinfused; fetal-cell contamination, RHD genotype, fetomaternal hemorrhage, and maternal antibodies not assessed; postpartum anti-D not given; and 3-month clinical follow-up too short to assess alloimmunization.
	11b	Discussion of the relevant medical literature.	Section 3 Discussion	Discussion cites relevant literature on PAS management, selective obstetric ICS, fetal red-cell contamination, alloimmunization risk, IABO, viscoelastic testing, anti-D prophylaxis, fetomaternal hemorrhage assessment, DEL phenotypes, and antibody follow-up (refs 1–19).
	11c	The rationale for your conclusions.	Section 3 Discussion; Section 4 Conclusion	Conclusions reflect the observed sequence of hemorrhage control, compatible allogeneic transfusion, reassessment of further red-cell need, and uncertain reinfusion risk. Inference is limited because salvaged blood was not reinfused, no comparator was available, and subsequent alloimmunization was not assessed.
	11d	The primary "take-away" lessons (without references).	Section 4 Conclusion	ICS preparation and reinfusion are distinct decisions. Non-reinfusion here was individualized and does not define a safe or preferable strategy or threshold. Future cases

				should use postpartum anti-D prophylaxis, validated fetomaternal hemorrhage quantification, and at least 6 months of maternal antibody follow-up. RHD genotyping may improve characterization, but its relevance to reinfusion is uncertain.
**Patient Perspective**	12	The patient should share their perspective on the treatment(s) they received.	Section 2.6 (final paragraph, 3-month follow-up)	Patient perspective is reported as part of the 3-month outpatient follow-up: the patient described her preoperative concerns about her rare blood type and possible massive transfusion, reported that the multidisciplinary consultations clarified the rationale for ICS preparation and the conditions for non-reinfusion, and expressed no regret about the non-reinfusion decision.
**Informed Consent**	13	The patient should give informed consent.	Section 2.4; Ethics Statement	Participation consent was not required. The manuscript reports separate written patient consent for publication of the case report and any potentially identifiable images or data.

Reference: Riley DS, Barber MS, Kienle GS, Aronson JK, von Schoen-Angerer T, Tugwell P, et al. CARE guidelines for case reports: explanation and elaboration document. *J Clin Epidemiol*. (2017) 89:218–35.
